# Synergistic Effects of Ternary Microbial Self-Healing Agent Comprising *Bacillus pasteurii*, *Saccharomyces cerevisiae,* and *Bacillus mucilaginosus* on Self-Healing Performance in Mortar

**DOI:** 10.3390/ma17194834

**Published:** 2024-09-30

**Authors:** Zhaoyun Wu, Jiaxuan Li, Tianlei Wang, Lei Zhang, Ben Peng, Changsheng Yue

**Affiliations:** 1 Reserch Institute of Urbanization and Urban Safety, School of Civil and Resource Engineering, University of Science and Technology Beijing, Beijing 100083, China; joeywu618@163.com; 2 School of Materials and Engineering, Tianjin Key Laboratory of Building Green Functional Materials, Tianjin Chengjian University, Tianjin 300384, China; lijiaxuan1215@163.com; 3 Central Research Institute of Building and Construction Co., Ltd., MCC Group, Beijing 100088, China

**Keywords:** ternary microorganism system, self-healing, mortar crack, microbially induced CaCO_3_ precipitation, synergic mineralization mechanism

## Abstract

In order to prevent structural damage or high repair costs caused by concrete crack propagation, the use of microbial-induced CaCO_3_ precipitation to repair concrete cracks has been a hot topic in recent years. However, due to environmental constraints such as oxygen concentration, the width and depth of repaired cracks are seriously insufficient, which affects the further development of microbial self-healing agents. In this paper, a ternary microbial self-healing agent composed of different proportions of *Bacillus pasteurii*, *Saccharomyces cerevisiae,* and *Bacillus mucilaginosus* was designed, and its crack repair ability was evaluated. When the mixing ratio was 7:1:2, the cell concentration was the highest, the precipitation amount of CaCO_3_ was the highest, and the crystallinity of calcite crystal was the highest. Compared to the single microorganism, the mortar specimens with ternary microorganisms had the largest repair area (up to 100%) and the deepest repair depth (CaCO_3_ presents at 9–12 mm from the crack surface). This is because when the concrete breaks, all three microorganisms are activated by water, O_2,_ and CO_2_. *Saccharomyces cerevisiae* and *Bacillus mucilaginosus* accelerated the growth of *Bacillus pasteurii* and more mineralized products; CaCO_3_ was rapidly formed and quickly filled on the crack surface. When CaCO_3_ seals the surface of the crack, the internal *Saccharomyces cerevisiae* and *Bacillus mucilaginosus* continue to play a role. *Bacillus mucilaginosus* can accelerate the dissolution of CO_2_ produced by the anaerobic fermentation of *Saccharomyces cerevisiae* and the hydrolysis of CO_3_^2−^, thereby improving the repair of the crack depth direction.

## 1. Introduction

Due to the inherent brittleness, low tensile strength, and complex service environment of concrete, cracking is almost inevitable. Traditional methods such as crack grouting and surface treatment are difficult and costly to effectively repair internal damage to concrete. The microbial self-healing technology for concrete is used to repair surface or internal microcracks in concrete that rely on their metabolism to obtain mineralized products (mainly calcite CaCO_3_), which has received extensive attention [1,2,3]. The green and environmentally friendly microbial self-healing technology can actively fill and repair cracks in concrete so as to achieve the purpose of maintaining concrete structures.

At present, microorganisms that induce CaCO_3_ precipitation through metabolic activity can be divided into the following categories: iron-reducing bacteria [4], denitrifying bacteria [5], nitrate-reducing bacteria [6], carbonic anhydrase bacteria [7], fungi [8], urease-producing bacteria [9], etc. Among the above microorganisms, many studies have focused on urease-producing bacteria (up to 84%) because they have good mineralization effects and can survive in a highly alkaline environment of concrete for more than 50 years [10,11]. *Bacillus pasteurii* (BP), which is a common urease-producing bacteria, can decompose urea to form a large amount of CO_3_^2−^, and then the precipitation of CaCO_3_ is formed via the interaction between CO_3_^2−^ and Ca^2+^, which has the characteristics of a fast mineralization rate and a high product yield. Rong et al. [12] found that when the microbial solution was added at 3% of the cement mass, the self-healing efficiency and impermeability ratio increased with the increase in BP concentration for cracks with larger width (0.6–0.7 mm), but the maximum self-healing efficiency was only 75.39 when the bacterial concentrations are 10^9^ cells/mL.

However, the mineralized products of most microorganisms are concentrated on the surface to block the crack surface, and it is difficult to develop in the depth direction of the crack, especially for cracks with larger widths. By adding a controlled oxygen-releasing tablet (ORT) containing CaO_2_ and lactic acid to concrete, the phenomenon of insufficient supply of deep oxygen can be alleviated, and the repair efficiency can be improved [13,14]. After 32 d of self-healing, the dissolved oxygen concentration in the solution of the specimens with ORT decreased from 15 mg/L to 4 mg/L, and about 27.5 mM of Ca^2+^ was precipitated. Only 6.9 mM of Ca^2+^ was precipitated in the specimens without ORT and almost 25% of the specimens with ORT [14]. Therefore, oxygen concentration is a key factor affecting the repair of aerobic microbial cracks. Anaerobic microorganisms can stably carry out microbial mineralization under low oxygen conditions to produce calcium carbonate, which can significantly improve the repair effect in the direction of crack depth [15,16]. Anaerobic denitrifying bacteria and aerobic alkalophilic Bacillus cohnii were loaded into expanded perlite and added to concrete. The crack repair width of this repair agent ranged from 0.1 to 0.7 mm [17]. Li et al. [18] successfully used BP and *Saccharomyces cerevisiae* (SC) binary microbial self-healing agents to repair cracks in mortar. It was found that when the microbial solution was added at 3% of the cement mass, and the mixing ratio of binary microorganisms was 6:4, the crack repair area rate was up to 97.1%, and biological calcium carbonate was generated at a distance of 9–12 mm from the surface of the sample. Therefore, the construction of an aerobic–anaerobic microbial system can effectively improve the width and depth of crack repair.

In addition, *Bacillus mucilaginosus* (BM) can produce carbonic anhydrase, which can catalyze the conversion of carbon dioxide into carbonate along the cracks and pores in the system, thereby accelerating its microbial mineralization rate [19,20]. The Brewers yeast and BM were loaded on the ceramsite carrier and then mixed into concrete. The maximum area repair rate could reach 87.5%, thus effectively improving the repair speed and depth of the cement-based materials [21]. Qian et al. studied the nucleation mechanism of bacteria during the formation of biological CaCO_3_ in detail by replacing Portland cement with C_3_S minerals. The incorporation of BM is conducive to the capture of carbon dioxide in the air, making it produce carbonates in pore or crack solutions and improving its mineralization efficiency. At the same time, the surface of BM is negatively charged, which can be used as a nucleation site to effectively adsorb calcium ions [22].

In order to improve the repair efficiency of a self-healing agent, especially the repair depth, a ternary microbial self-healing agent was prepared by using BP, SC, and BM in different proportions. According to the growth law and mineralization of microorganisms, the optimum ratio of the ternary microbial self-healing agent was determined, and it was applied to the repair cracks to evaluate its repair effect. At the same time, the morphology and composition of the mineralized products were analyzed by scanning electron microscopy (SEM), X-ray diffraction (XRD), and thermogravimetric analysis (TG) to determine the mineralization efficiency so as to reveal the mechanism of ternary microbial synergistic mineralization.

## 2. Materials and Methods

### 2.1. Microorganisms and Culture Medium

In this work, *Bacillus pasteurii* (BP, DSM 33) was provided by the German Collection of Microorganisms and Cell Cultures. *Saccharomyces cerevisiae* (SC, 2.3095) was purchased from the China General Microbiological Culture Collection Center. A kind of *Bacillus mucilaginosus* (BM, GLRT202Ca) purchased from the Institute of Karst Environment and Rocky Desertification Control in Southwest China was selected as the carbonic anhydrase-producing bacteria in this system. The cells used were also rods with rounded ends, 1.0–1.5 μm in diameter and 4.0–7.0 μm in length. These three microorganisms can survive and maintain high activity at pH values up to 12.5, 12, and 12.5 through alkaline-resistance selection, respectively.

The nutrients are fully dissolved in water, placed in a high-pressure sterilizer for sterilization, and then cooled to room temperature to prepare an aqueous solution of nutrients (Table 1). Subsequently, a urea solution (4 mol/L) with a volume concentration of 4% was added to the nutrient solution, and the pH of the nutrient solution was adjusted to 7.0 using a 2 mol/L NaOH aqueous solution [23].

### 2.2. Preparation of Ternary Microorganism Self-Healing Agent

**Figure 1:**
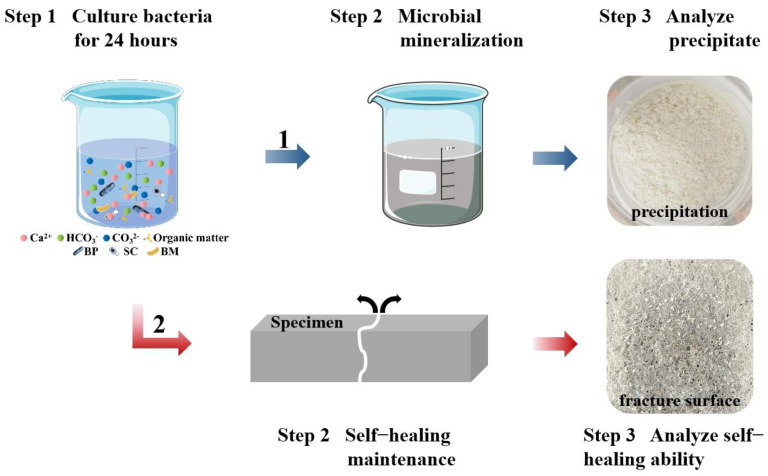
The flow chart of ternary microbial self-healing agent on self-healing performance in mortar.

The flow chart of ternary microbial self-healing agent on self-healing performance in mortar is shown in Figure 1. The preparation method of ternary microbial self-healing agent includes three steps. Firstly, the three types of microorganisms were placed in a shaking incubator (ZDP-250, Shanghai Jinghong Experimental Co., Ltd., Shanghai, China) at 170 rpm and 33 °C for 24 h to ensure a cell concentration of 10^9^ cells/mL. Secondly, the microorganism suspensions were mixed according to a certain ratio to obtain a BP-SC-BM mixture suspension. The mixing ratios (volume ratio of BP suspension, SC suspension, and BM suspension) were 8:1:1, 7:2:1, 7:1:2, 6:3:1, 6:2:2, 6:1:3, 5:4:1, 5:3:2, 5:2:3, and 5:1:4, respectively. In addition, three single-type microorganism suspensions with mixing ratios of 10:0:0, 0:10:0, and 0:0:10 were prepared, representing the microorganism suspensions with only BP, SC, and BM, respectively. Finally, 5 mL of the obtained BP-SC-BM mixture microorganism suspension was added to a 95 mL culture medium and cultured for 24 h for subsequent evaluation of physiological and biochemical characteristics and crack repair effects.

### 2.3. Microbial Mineralization

In this study, an equal volume of urea (2.0 mol/L), glucose (2 wt%), calcium acetate (2.0 mol/L), and microbial suspension were uniformly mixed, in which urea, glucose, and calcium acetate were used as substrates to produce precipitates [23]. Two mineralization conditions were set to simulate the mineralization environment in concrete cracks. The experiment was conducted in ordinary air environments, simulating cracks under oxygen-rich conditions. The other was the oxygen-poor condition, which was realized in a vacuum-drying furnace and could simulate the case of deep cracks [24]. After mixing the substrates with the microbial suspension in a beaker, the beaker was immediately placed in a vacuum drying furnace, the air inside the furnace was evacuated, and the mineralization process was started under these anaerobic conditions. The mixed solution was placed in a beaker and mineralized for 72 h under different conditions. The mass of the precipitate was weighed to calculate the microbial mineralization rate.

### 2.4. Preparation of Mortar Specimens and Cracks Creation

The specimens used in this experiment were mortar specimens with dimensions of 40 × 40 × 160 mm. By mixing ordinary Portland cement, standard sand, tap water, microorganism suspension, and substrate, the mixing proportions were as shown in Table 2 and Table 3. To quantitatively study the effect of microbial mixing ratios on crack self-healing, the microorganism suspension was added directly to the mortar, and the weight of the substrate was equal to 3% of the cement. A larger water–cement ratio (*w*/*c* = 0.5) was used to ensure the survival of the microorganisms [25,26]. After curing for 24 h, the molded specimens were kept at 20 °C and humidity greater than 90% for 7 d, and then the cracks were made with an average width of 0.4 mm through the three-point bending test. The molding and curing methods are based on GB/T 29756-2013 “Physical test method of dry mixed mortar”.

### 2.5. Characterization Methods

#### 2.5.1. The pH Value and Cell Concentration of Microorganism Suspensions

We measured the pH value of microbial suspension using a PHS-25 precision acidimeter with an accuracy of 0.1 (Shanghai Leithi Instrument Co., Ltd., Shanghai, China) to monitor the metabolic status of microorganisms during their growth process.

Due to the significant differences in cell size among the three microorganisms, the traditional method of measuring OD600 using a UV spectrophotometer is not applicable. In this study, the Neubauer counting chamber was used to investigate the growth law of multiple mixed microorganisms [26,27]. The microorganism suspensions with different inoculation times (0–36 h) were dropped into the Neubauer counting chamber, and we calculated the number of live cells using a super depth 3D microscope system (VHX-600, KEYENCE, Osaka, Japan). To reduce the effect of uneven distribution of microbial suspensions, two samples of the upper microorganism suspension were taken simultaneously, and the average of the cell concentrations was also taken. The formula for calculating the concentration of cells (cells/mL) is as follows:

(1) $$y=\frac{25ax}{5}\times 10^{4}$$

where *y* is total cell concentrations per mL, *x* is the number of live cells in the counting chamber, and *a* is the dilution factor.

#### 2.5.2. Microbial Mineralization Products

Two methods were used to determine the amount of the precipitate after the mineralization process. The change in Ca^2+^ concentration can be used to characterize the settling speed of the precipitate. Therefore, the EDTA titrimetric method was used to determine the calcium ion concentration in the supernatant at different times (1 h, 3 h, 6 h, 24 h, 48 h, and 72 h) after mixing microbial suspension with substrate [28].

The amount of precipitation can be determined using the acid-washing method [29]. To ensure the completion of the microbial mineralization reaction, 20 mL of HCl solution (in excess amount) with a concentration of 2.5 mol/L was added to the microbial suspension inoculated for 72 h. When the precipitate is completely dissolved, the solution before and after the reaction is weighed. The amount of CaCO_3_ precipitate is calculated as follows:

(2) $$m_{{CaCO}_{3}}=\frac{M_{{CaCO}_{3}}}{M_{{CO}_{2}}}\times m_{{CO}_{2}}$$

where $m_{{CaCO}_{3}}$ is the mass of CaCO_3_ precipitate, $m_{{CO}_{2}}$ is the difference in the mass of the solutions before and after the acid washing, $M_{{CaCO}_{3}}$ is the molar mass of CaCO_3_, and $M_{{CO}_{2}}$ is the molar mass of CO_2_.

After 72 h of microbial mineralization precipitation, the precipitate was collected and dried in an oven at 80 °C. The crystalline phase of the mineralization product was analyzed by X-ray Diffraction (XRD, JN-210, Rigaku, Tokyo, Japan) with a scanning voltage of 40 kV, a step size of 0.02°, and a scan range of 10°–80°. The microstructures of the precipitates were observed with a scanning electron microscope (SEM, JSM-7800F, JEOL, Tokyo, Japan).

#### 2.5.3. Crack Self-Healing Ability of Ternary Microbial Self-Healing Agent

The cracked specimen was taken out, and the self-healing effects of the crack surface and depth were observed, according to the standard “Technical specification for application of microbial-based self-healing concrete” (T/CECS 973-2021). Firstly, use a digital camera to capture images of cracks before and after self-healing, and use image processing software (Image J 1.53t) to process the images. By setting grayscale values and calculating the number of pixels, the area change in the crack surface is reflected. The percentage of self-healing area is calculated as follows:

(3) $$area\;self-healing\;percentage=\frac{{Area}_{0}-{Area}_{28}}{{Area}_{0}}$$

where ${Area}_{0}$ is the area of the crack before healing (0 d), and ${Area}_{28}$ is the area of the crack after healing for 28 d.

Secondly, the specimen was opened along the crack, one of the fracture surfaces was taken, and the powder samples were collected from the crack surface to the inside of the crack with the file. Afterward, the powder samples was analyzed by SEM and TG analyzer. After grinding a certain amount of powder, the remaining thickness was measured by a vernier caliper, and the interval range of the powder in the crack depth direction was calculated to analyze the distribution of CaCO_3_ in the fracture depth direction.

## 3. Results and Discussion

### 3.1. Optimal Mixing Ratio to Ternary Microorganism

#### 3.1.1. Growth Law of Ternary Microorganism System

**Figure 2:**
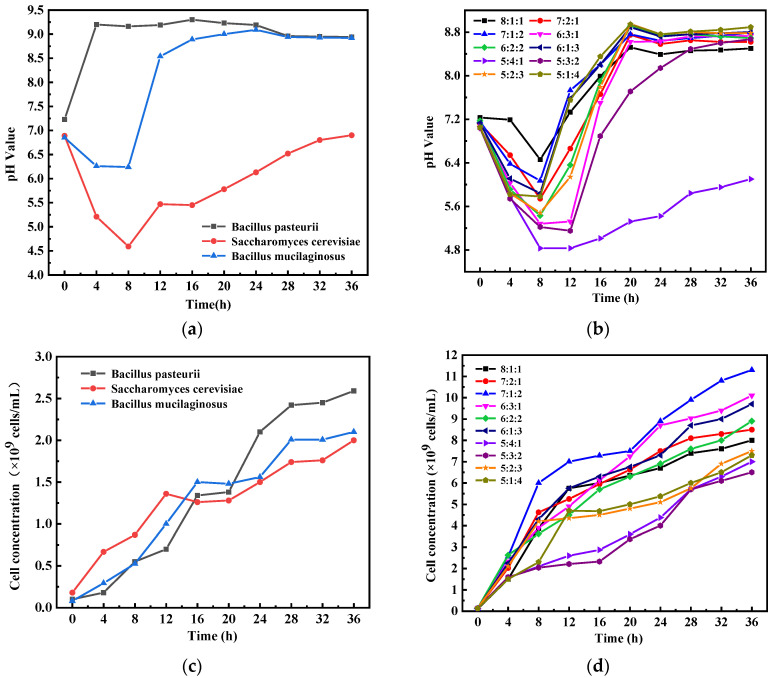
Change in pH value of each microorganism suspension (**a**) and microorganism suspension with different mixing ratios (**b**); Changes in cell concentration of each microbial suspension (**c**) and microorganism suspension with different mixing ratios (**d**).

The growth law of the microorganisms in the ternary microorganism system could be characterized by the evolution of the pH value of the microorganism suspension and the change in the cell concentration during the co-culturing period. Different pH changes are associated with microbial metabolites and metabolic rates. It can be seen from Figure 2a that the pH of BP increased rapidly to about 9 during the growth process and then remained stable. The reason is that the urease produced by metabolism decomposes urea to produce a large amount of OH^−^, which increases the pH of the microbial suspension. The pH of SC drops rapidly to about 4.5 due to the breakdown of glucose to produce CO_3_^2−^ at first. With the prolongation of culture time, the alkaline metabolites produced by microorganisms slowly increase the pH of the microbial suspension to about 6.5 [30]. The pH of BM decreased to about 6.25 at first and then increased to more than 8.5, which was similar to that of SC.

It can be seen from Figure 2b that the pH of all mixed microbial suspensions decreased at first and then increased. The decrease in pH is due to an increase in CO_3_^2−^ in the microbial suspension, which is due to the metabolism of SC and BM. Subsequently, due to the decomposition of urea by BP to produce OH^−^ and a large number of alkaline metabolites produced by microorganisms, the pH value of the microorganism suspension increased again. At the same time, the greater the proportion of SC in the ternary microorganism system, the greater the decrease in pH. When the mixing ratio was 5:4:1, the pH value could not rise to more than 7. The acidic environment can hardly provide a favorable environment for microorganisms to induce CaCO_3_ precipitation. All other mixing ratios of the ternary microorganism system can increase the pH value to more than 8.5.

Figure 2c shows that the cell concentration of the three microorganisms increased to 1.5–2.5 × 10^9^ cells/mL after 36 h of inoculation. As can be seen from Figure 2d, the cell concentration can rise to more than 6 × 10^9^ cells/mL in a ternary microorganism system, reaching more than twice the concentration of individual microorganisms. When the inoculation amount of BP was 80% and 50%, the increase in the cell concentration of the bacterial suspension was not as much as that of the inoculation amount of 70% and 60%. The addition of an appropriate amount of SC and BM can improve the microbial activity of BP. When the mixing ratio was 8:1:1, the proportion of SC and BM was small, and the promoting effect on the growth of BP was not significant. When BP accounted for 50%, the decrease in pH in the early stage of microbial growth had an adverse effect on the growth of microorganisms. However, when BP accounted for 70% and 60%, the addition of an appropriate amount of SC and BM provided organic nitrogen, amino acids, minerals, and peptides, which promoted BP’s microbial activity and growth. Therefore, from the concentration of cells alone, the optimal mixing ratio is 7:1:2.

#### 3.1.2. Microbial-Induced CaCO_3_ Precipitation

**Figure 3:**
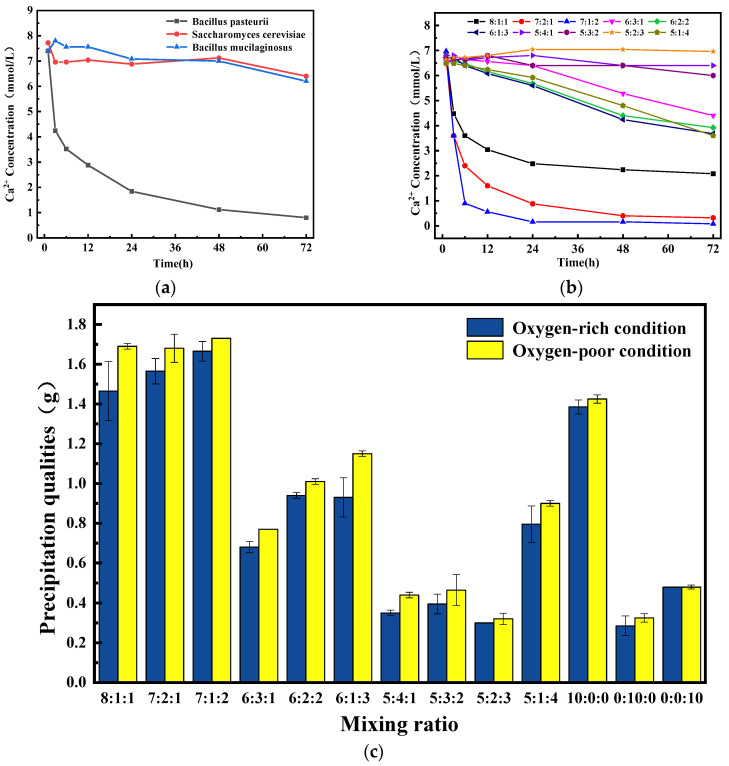
Concentration of Ca^2+^ in the supernatant of microorganism–substrate solution during mineralization of individual microorganism (**a**) and the ternary microorganisms (**b**) under the oxygen-rich condition; amount of CaCO_3_ precipitate obtained after mineralization for 72 h (**c**).

It is necessary to study its mineralization process because the mineralization caused by microbial activity directly affects the repair effect of concrete cracks. Therefore, the optimal mixing ratio can be determined according to the amount of mineralization products and the sedimentation rate. Figure 3a shows the concentration of Ca^2+^ in the supernatant of the microbial–substrate solution during the mineralization of individual microorganisms under oxygen-rich conditions. Compared with the other two microorganisms, BP has a faster settling speed of the precipitate. Figure 3b shows the concentration of Ca^2+^ in the supernatant of a ternary microorganism system with different mixing ratios during mineralization under the same conditions. When the percentage of SC exceeds 20%, the concentration of Ca^2+^ in the solution remains almost unchanged. When the percentage of BM exceeded 20%, the concentration of Ca^2+^ in the supernatant decreased very slowly. When the BP was 80% and 70%, the decrease rate of Ca^2+^ concentration in the precipitation solution decreased rapidly in the first 12 h and decreased very slowly after 12 h.

Figure 3c shows the amount of precipitation after mineralization of different microbial mixtures under two mineralization conditions. It can be seen that the amount of precipitate produced under oxygen-poor conditions is higher than that under the same proportion of oxygen-rich conditions for all the mixing ratios. Because there is no loss of CO_2_ which is produced by SC in the vacuum drying oven, increasing the availability of more soluble CO_2_ for BM. For individual microorganisms, the best mineralization effect is BP (mixed ratio of 10:0:0), and the amount of mineralization products is about 3 times that of the other two microorganisms. Since the CO_2_ produced by SC cannot be hydrolyzed in large quantities, the number of mineralized products produced by BM (mixing ratio of 0:0:10) is relatively small. The content of CO_2_ in the air is low, resulting in the least number of mineralized products produced by SC (mixing ratio of 0:10:0). In the ternary microorganism system, the precipitation amount at the BP proportion of 80% and 70% is higher than that being produced at the BP proportion of 60% and 50%. When the proportion of BP in the microorganism suspension is the same, the more the proportion of SC, the less the precipitation was generated. The reason is that part of the carbon dioxide produced by SC metabolism is dissolved in the solution, making the solution acidic, thereby inhibiting the growth of BP. It is worth noting that microbial mineralization produced the most CaCO_3_ when the mixing ratio was 7:1:2.

#### 3.1.3. Composition and Microstructures of Microbial Mineralization Products

**Figure 4:**
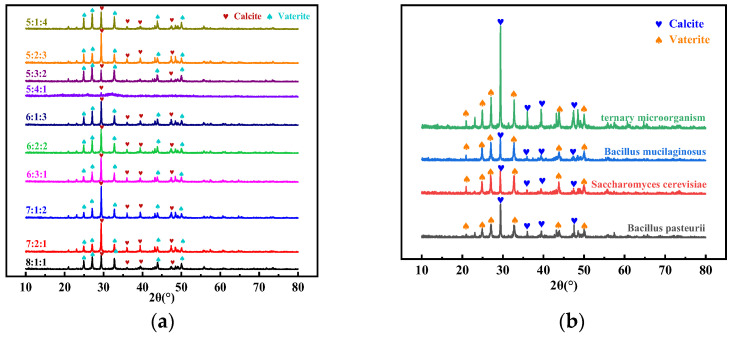
XRD patterns of mineralization products with different mixing ratios (**a**) and different microorganisms (**b**).

Figure 4a shows the XRD patterns of the precipitate products at different mixing ratios in ternary microorganisms, showing that the precipitates are calcite and vaterite forms of CaCO_3_. When the mixing ratio is 7:1:2, the diffraction peak of calcite is sharpest, indicating that the precipitate produced has good crystallinity. Compared to the XRD pattern of mineralization products from individual microorganisms, the diffraction peak intensity of mineralization products in the ternary microorganism system with a mixing ratio of 7:1:2 is significantly higher, indicating better crystallinity and once again proving the advantages of the ternary microorganism system (Figure 4b).

**Figure 5:**
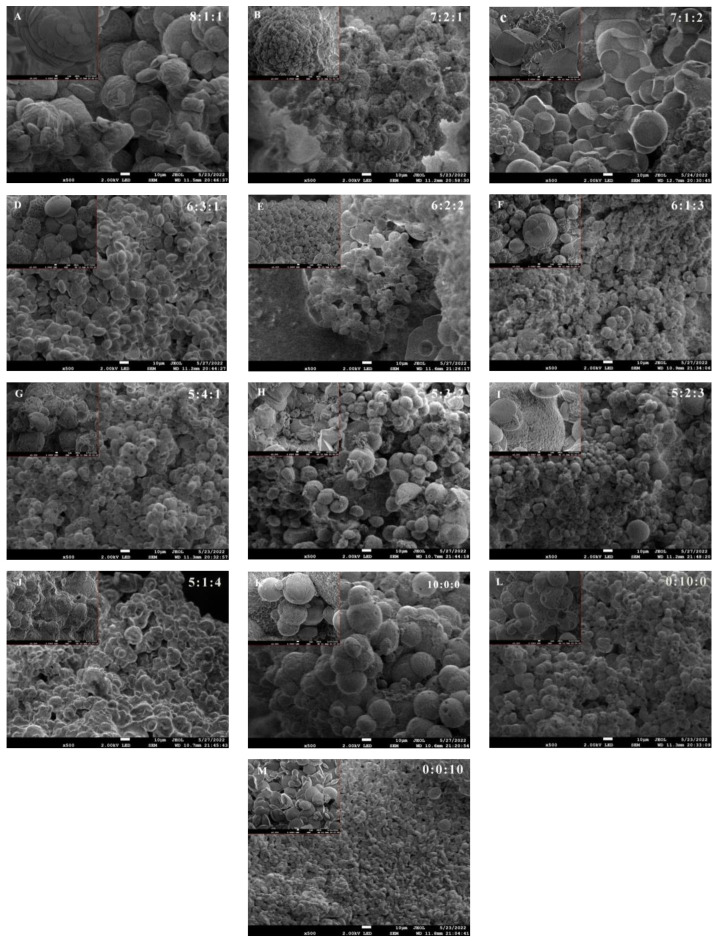
SEM of mineralization products with different mixing ratios.

The SEMs of mineralization products at different mixing ratios of microorganisms are shown in Figure 5, from which it can be seen that the mineralization products of microorganisms are formed by the accumulation of cubic, spherical, and ellipsoidal shape CaCO_3_ crystal particles, but the morphologies of precipitates at different mixing ratios are different. The mineralization products of BP are cubes and spherical particles stacked into a spherical structure, but the structure is not dense. The mineralization products of BM are the loose accumulation of small ellipsoid particles, and the particles are dense. The mineralization product of SC is the loose accumulation of spherical small particles, and there are many pores on the particles.

When BP accounts for 80%, the mineralization products show a spherical structure with ellipsoidal particles accumulated, and the structure is not dense. When BP accounts for 70%, the CaCO_3_ particles constituting the product change from ellipsoidal to cubic and spherical. When the mixing ratio is 7:1:2, the mineralization product has a dense structure, and the cubic and spherical CaCO_3_ particles that make up the structure have a larger and more regular shape. When BP accounts for 60% and 50%, the size of CaCO_3_ particles is smaller. As the percentage of SC increases, CaCO_3_ particles become sparser, and as the percentage of BM increases, CaCO_3_ particles become more irregularly ellipsoidal.

### 3.2. Self-Healing of Cracks with Ternary Microorganism System

#### 3.2.1. Percentage of Repair Surface Area

**Figure 6:**
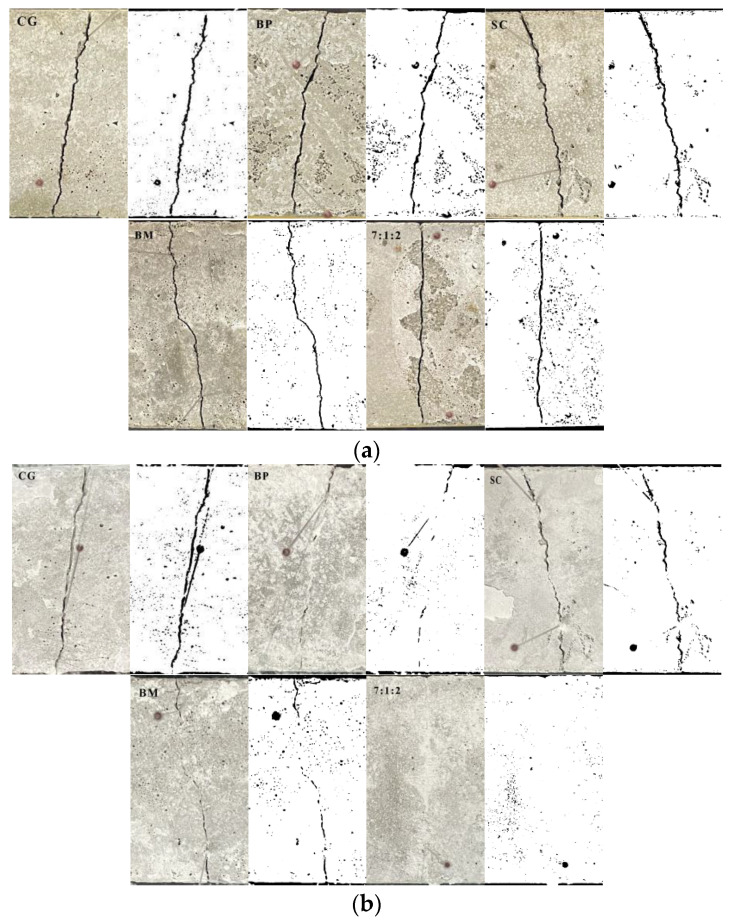
Digital photos and binary images of mortar cracks before (**a**) and after (**b**) self-healing.

After 28 d of self-healing, all the cracks were self-repaired to a certain extent (Figure 6). The percentage of repair area of the control specimen without adding microorganisms (CG) was 4.3%, and the percentage of repair area of the mixed ratio of 10:0:0, 0:10:0, 0:0:10, and 7:1:2 was 80.8%, 18.7%, 39.8%, and 100%, respectively. Compared with the degree of repair surface, the self-healing effect of the ternary microorganism (7:1:2) was the best after 28 d; the crack surface can be completely repaired.

#### 3.2.2. Effect of Self-Healing Cracks at the Depth

**Figure 7:**
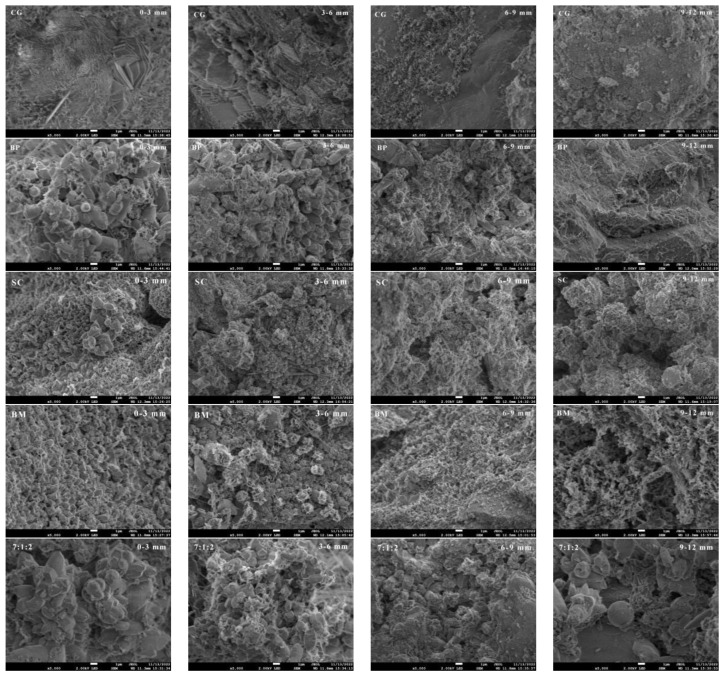
SEM of powders in different crack depths.

Figure 7 shows the SEM of the powders at different crack depths of the five groups of specimens. The specimens without the addition of microorganisms (CG) had very little CaCO_3_ at different crack depths, and there are a large number of hydration products, including flocculants C-S-H and regular hexagonal Ca(OH)_2_, etc. The CaCO_3_ in the BP specimens were regular and dense at 0–3 mm from the crack surface, sparse at 3–6 mm, less CaCO_3_ at 6–9 mm, and almost no CaCO_3_ at 9–12 mm. The CaCO_3_ shapes of SC specimens at 0–6 mm from the crack surface were small cubic shapes, and the size became smaller at 6–12 mm. The shapes of CaCO_3_ in BM specimens were also small and cubic at 0–3 mm from the crack surface, while the size became smaller at 3–6 mm and basically free of CaCO_3_ at 6–12 mm. In the ternary microbial specimens (7:1:2), a large number of dense CaCO_3_ with relatively large size appeared at 0–3 mm from the crack surface, while CaCO_3_ was small but tightly packed at 3–9 mm and became sparse at 9–12 mm. Importantly, only the specimens repaired by ternary microbial self-healing agent still had CaCO_3_ crystals at 9–12 mm.

**Figure 8:**
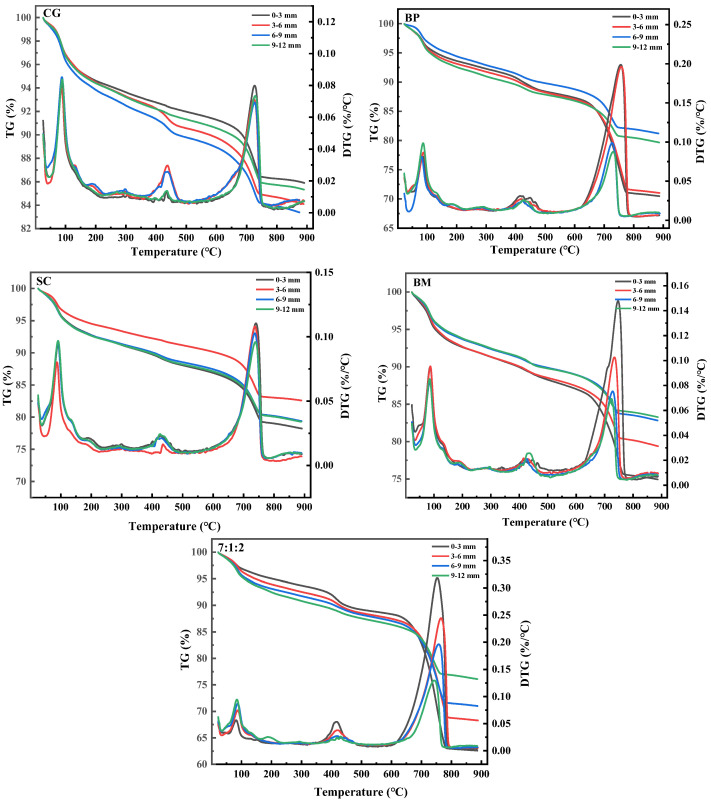
TG/DTG curves of powders with different crack depths.

The powder at 0–3, 3–6, 6–9, and 9–12 mm from the crack surface was taken to analyze the effect of different mixing ratios on the depth self-healing effect (Figure 8). The loss at 0–100 °C is the mass of crystal water in the sample, the loss at 400–500 °C is the mass of Ca(OH)_2_, and the loss at 600–800 °C is the mass of CaCO_3_. A certain amount of CaCO_3_ exists even in the control specimen (CG), which should be attributed to the chemical reaction between the Ca(OH)_2_ in the mortar and the CO_2_ that penetrates into the specimen through the cracks. In contrast, the depth of repaired concrete cracks by ternary microorganisms was the largest, with 11.98% of CaCO_3_ at a distance of 9–12 mm from the surface. The weight loss percentage of CaCO_3_ in the specimen repaired by the ternary microbial self-healing agent with a mixing ratio of 7:1:2 was 2.90 times, 2.26 times, 1.72 times, and 1.10 times higher than that of the control specimen at the depths of 0–3 mm, 3–6 mm, 6–9 mm, and 9–12 mm, respectively. It is worth noting that the weight loss percentages of CaCO_3_ in specimens repaired by the ternary microbial self-healing agent were 28.8%, 38.1%, 95.7%, and 106.6% higher at 0–3 mm, 3–6 mm, 6–9 mm, and 9–12 mm depths, respectively, compared to a single microbial system consisting only of BP. In addition, compared with the single microbial remediation agent, the content of CaCO_3_ in the precipitation of each depth of the ternary microbial remediation agent was the highest. Above all, the ternary microbial self-healing agent can produce more mineralized precipitates to fill the cracks, indicating the self-healing effect of mortar cracks mixed with ternary microorganisms is better. Based on the above results, it is considered that the ternary microbial self-healing agent with a mixture ratio of 7:1:2 is more suitable as a concrete self-healing agent than the monomer microbial self-healing agent.

In order to better understand the mineralization efficiency of the ternary microbial system and the self-healing efficiency of artificial crack mortar specimens, it is necessary to clarify the mineralization mechanism of the ternary microbial system. When concrete cracks, water, and oxygen can enter the interior of the concrete through the cracks. At the same time, BP, SC, and BM in the ternary microbial system can be activated. An appropriate amount of SC and BM can promote the propagation of BP, which can rapidly decompose the urea pre-added to the concrete mix as the substrate of the mineralization process, generating a large amount of CO_3_^2−^. The CO_3_^2−^ reacts with the Ca^2+^ in concrete to form CaCO_3_, which mainly accumulates on the crack surface. This process is similar to that of a single microbial system, such as using BP alone as a self-healing agent when cracks are shallow.

When the surface of the concrete crack is covered by rapidly generated CaCO_3_ precipitates, the ability of BP to degrade urea weakens under anaerobic conditions, such as in the deeper parts of the crack. In this case, SC and BM play an indispensable role. SC can continuously produce CO_2_ by decomposing glucose, while BM produces carbonic anhydrase, which accelerates the hydrolysis of CO_2_ generated by SC into carbonate ions, promoting the mineralization process and enhancing the repair effect in the deeper parts of the crack. This method solves the problem of excessive CO_2_ produced by SC that cannot be hydrolyzed and the potential structural defects that may occur during gas emission. Therefore, the appropriate mixing ratio in the ternary system, such as 7:1:2, is of great significance for the ternary microbial self-healing agent.

## 4. Conclusions

In this study, a ternary microbial self-healing agent for microbial concrete cracks consisting of BP, SC, and BM was successfully prepared. When the mixing ratio of BP, SC, and BM is 7:1:2, the microbial concentration and precipitation are highest. At the same time, due to the influence of microbial metabolism and the pH value of microbial solutions, mineralized products CaCO_3_ have the best crystallinity at this ratio. Compared with the mineralization and remediation effects of a single microorganism, ternary microorganisms have superior effects. This is mainly the anoxic environment inside the crack. SC anaerobically decomposed glucose to produce CO_2_, and BM accelerated the hydrolysis of CO_2_, providing CO_3_^2−^ for the depth of the crack, which was conducive to deep repair. Therefore, the development of this self-healing agent provides some useful guidance for solving the problem of low repair depth. However, the influence of different environmental factors on the repair effect of ternary microbial remediation agents, the durability of the repaired sample, and its long-term repair effect need to be further studied in the future.

## Figures and Tables

**Table 1:** Nutrients in the culture medium (g/L).

Beef Extract	Tryptone	Glucose	Yeast Extract	Na_2_HPO_4_	KCl	Sucrose	(NH_4_)_2_SO_4_	MgSO_4_
3.00	15.00	10.00	5.60	5.00	0.28	20.00	1.04	1.01

**Table 2:** Proportion of microbial self-healing mortar.

Cement/g	Water/g	Sand/g	Microorganism Suspension/mL	Substrates/g
650.00	300.00	1300.00	25.00	21.02

**Table 3:** Composition of substrates (g).

Calcium Acetate	Urea	Beef Extract	Tryptone	Glucose	Yeast Extract	Na_2_HPO_4_	KCl	(NH_4_)_2_SO_4_	MgSO_4_
15.00	2.90	0.24	1.14	0.75	0.42	0.39	0.02	0.08	0.08

## Data Availability

The original contributions presented in the study are included in the article, further inquiries can be directed to the corresponding author/s.
